# Marine Biodiversity in Juan Fernández and Desventuradas Islands, Chile: Global Endemism Hotspots

**DOI:** 10.1371/journal.pone.0145059

**Published:** 2016-01-06

**Authors:** Alan M. Friedlander, Enric Ballesteros, Jennifer E. Caselle, Carlos F. Gaymer, Alvaro T. Palma, Ignacio Petit, Eduardo Varas, Alex Muñoz Wilson, Enric Sala

**Affiliations:** 1 Pristine Seas, National Geographic Society, Washington, District of Columbia, United States of America; 2 Fisheries Ecology Research Lab, University of Hawaii, Honolulu, Hawaii, United States of America; 3 Millennium Nucleus for Ecology and Sustainable Management of Oceanic Islands (ESMOI), Coquimbo, Chile; 4 Centre d'Estudis Avançats (CEAB-CSIC), Blanes, Spain; 5 Marine Science Institute, University of California Santa Barbara, Santa Barbara, California, United States of America; 6 Universidad Católica del Norte, Coquimbo, Chile; 7 Centro de Estudios Avanzados en Zonas Áridas, Coquimbo, Chile; 8 Instituto de Ecología y Biodiversidad, Coquimbo, Chile; 9 FisioAqua, Santiago, Chile; 10 OCEANA, SA, Santiago, Chile; The Australian National University, AUSTRALIA

## Abstract

The Juan Fernández and Desventuradas islands are among the few oceanic islands belonging to Chile. They possess a unique mix of tropical, subtropical, and temperate marine species, and although close to continental South America, elements of the biota have greater affinities with the central and south Pacific owing to the Humboldt Current, which creates a strong biogeographic barrier between these islands and the continent. The Juan Fernández Archipelago has ~700 people, with the major industry being the fishery for the endemic lobster, *Jasus frontalis*. The Desventuradas Islands are uninhabited except for a small Chilean military garrison on San Félix Island. We compared the marine biodiversity of these islands across multiple taxonomic groups. At San Ambrosio Island (SA), in Desventuradas, the laminarian kelp (*Eisenia cokeri*), which is limited to Desventuradas in Chile, accounted for >50% of the benthic cover at wave exposed areas, while more sheltered sites were dominated by sea urchin barrens. The benthos at Robinson Crusoe Island (RC), in the Juan Fernández Archipelago, comprised a diverse mix of macroalgae and invertebrates, a number of which are endemic to the region. The biomass of commercially targeted fishes was >2 times higher in remote sites around RC compared to sheltered locations closest to port, and overall biomass was 35% higher around SA compared to RC, likely reflecting fishing effects around RC. The number of endemic fish species was extremely high at both islands, with 87.5% of the species surveyed at RC and 72% at SA consisting of regional endemics. Remarkably, endemics accounted for 99% of the numerical abundance of fishes surveyed at RC and 96% at SA, which is the highest assemblage-level endemism known for any individual marine ecosystem on earth. Our results highlight the uniqueness and global significance of these biodiversity hotspots exposed to very different fishing pressures.

## Introduction

In 1574, the Spanish explorer Juan Fernández was seeking a faster route between Callao, Perú and Valparaíso, Chile. By detouring west from the coast he managed to avoid the north-flowing Humboldt Current, and in the process discovered the uninhabited Juan Fernández and Desventuradas islands off the coast of what is now Chile [1]. These remote islands are among the few oceanic archipelagos along the west coast of South America, yet our scientific understanding of them is extremely limited [2–7]. The Juan Fernández Archipelago lies ~ 600 km west of Valparaiso, Chile and consists of three islands: Robinson Crusoe (also known as Isla Más a Tierra), Santa Clara, and Alejandro Selkirk (also known as Isla Más Afuera) [8–9]. Robinson Crusoe Island is best known from the novel by Daniel Defoe that bears its name, and is based on the true story of the Scottish sailor, Alexander Selkirk, who spent more than four years alone on the island before being rescued in 1709 [8]. These islands were once the domain of pirates and privateers, and later were of strategic important to a number of nations [10]. Located 750 km north of the Juan Fernández Archipelago are the Desventuradas Islands, which are ~ 900 km west of Antofagasta, Chile, and consist of the islands of San Ambrosio and San Félix, along with several small islets [2].

Due to their extreme isolation, these islands are known to have a high degree of endemism in both their terrestrial and marine biota, as well as a unique mix of tropical, subtropical, and temperate marine species [7, 11–13]. Although relatively close to mainland South America, the Juan Fernández biogeographic province is considered a distinct ecoregion, with a strong southwest Pacific component to its marine fauna [14–15]. Taxonomy and biogeography of the coastal fishes of Desventuradas and Juan Fernández islands indicate a Western Pacific origin, and they therefore represent the easternmost extension of the Indo-West Pacific biogeographic region [13, 16]. Biogeographic affinity with the Tropical Eastern Pacific is almost negligible despite geographic proximity comparable to the central Pacific [17].

Endemic hotspots are vital to our understanding of speciation and the origins and maintenance of biodiversity, and as a result they have extremely high conservation value [18–20]. Marine centers of endemism predominate in places isolated by geography or oceanography. For example, isolated islands rich in endemics include Mauritius and La Reunion in the Indian Ocean, Hawaii, Easter Island, and the Marquesas in the Pacific, and St. Helena and Ascension Islands in the Atlantic [21–22]. The Juan Fernández Archipelago was designated as a national park by the government of Chile in 1935, and a UNESCO Biosphere Reserve in 1977. These islands are referred to as Chile’s Galapagos, and like the Galapagos they are of volcanic origin (3–6 Mya) and are characterized by geographic isolation, high endemism, and charismatic fauna such as the Juan Fernandez fur seal (*Arctocephalus philippii*), Magellanic Penguins (*Spheniscus magellanicus*), and the only known breeding populations of two petrel species, Stejneger's Petrel (*Pterodroma longirostris*) and the Juan Fernandez Petrel (*Pterodroma externa*), which are both listed as vulnerable by IUCN [9]. De Filippi's Petrel (*Pterodroma defilippiana*) is also listed by IUCN as vulnerable and is only known to breed on the Desventuradas Islands, and possibly Juan Fernández [23–24].

The fishery for the endemic lobster (*Jasus frontalis*), which has been locally exploited since 1893 [25], is the main source of economic revenue to the local economy of Juan Fernández, and although the abundance and distribution of this species has declined over time, co-management of the fishery has resulted in relatively sustainable catches in recent years [26–27]. An informal management system exists whereby trap location is governed by a complex, highly structured system with high compliance [28]. A number of nearshore fish species are targeted as bait for the lobster fishery, as well as for local consumption [29–30]. On a regular basis fishermen from Juan Fernández seasonally travel to Desventuradas Islands to catch lobsters [2, 29].

The objectives of this research were to compare the nearshore marine biodiversity of Juan Fernández and Desventuradas islands, assess their importance for global marine biodiversity, and assess how contrasting fishing pressure may affect marine ecosystems, even in remote locations.

## Materials and Methods

### Ethics statement

Data were collected by all authors in collaborative partnerships. Non-invasive research was conducted, which included photographs and visual estimates described in the methods. The Chilean Navy and the Undersecretary of Fishing granted all necessary permission and permits to conduct this research. No vertebrate sampling was conducted and therefore no approval was required by the Institutional Animal Care and Use Committee. S1 Table contains the GPS coordinates for all research sites.

### Locations

**Fig 1:**
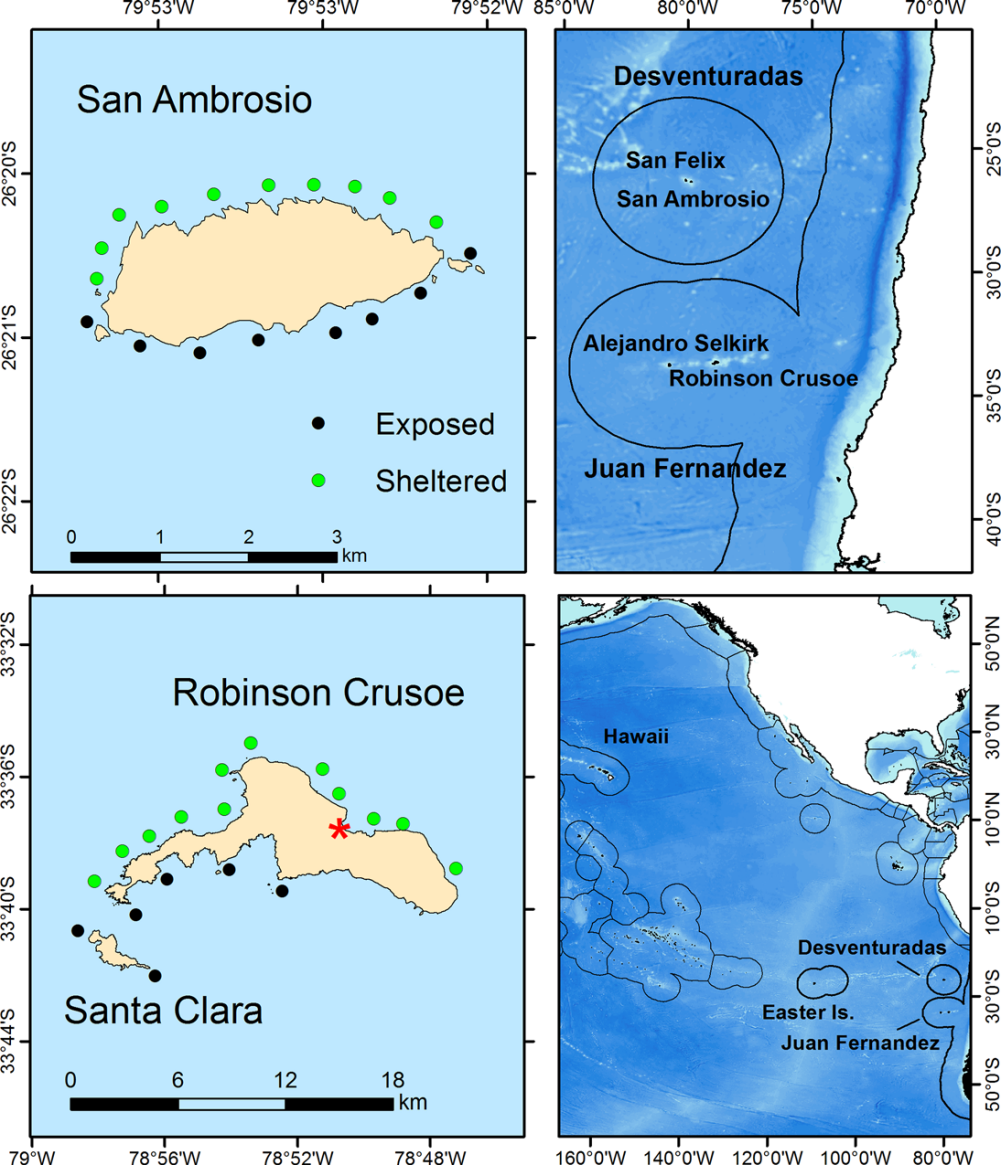
Sampling sites by wave exposure around Robinson Crusoe and Santa Clara islands in the Juan Fernández Archipelago and San Ambrosio in Desventuradas Islands. The town of San Juan Bautista on Robinson Crusoe Island is denoted by the red asterisk. Location of the islands in relation to the coast of Chile are shown in the upper right panel. Black lines are Chile’s Exclusive Economic Zones around the islands. The lower right panel shows the locations of these islands in the broader Pacific. Pacific basemaps are ETOPO1 from NOAA’s National Geophysical Data Center [34].

The Desventuradas Islands are part of a chain of seamounts and submarine volcanoes that extends westward along the Nazca Ridge and includes Salas y Gómez and Easter islands, while the Juan Fernández Archipelago lies on a much shorter chain of seamounts ~ 750 km to the south (Fig 1) [31–33]. Between 2004 and 2014, sea surface temperature averaged 19.0°C (range: 15.6–22.85) at Desventuradas and 17.1°C (range: 12.8–21.5) at Juan Fernández [34]. At Desventuradas there is a small Chilean military garrison on San Félix Island, while San Ambrosio is uninhabited. The vast major of the 700 inhabitants of the Juan Fernández Archipelago live in the town of San Juan Bautista, with a small resident population (~50 people) at Alejandro Selkirk Island, 170 km to the west. At Deventuradas, we surveyed San Ambrosio Island, and in the Juan Fernandez group we surveyed Robinson Crusoe and Santa Clara islands.

### Sample design

Using SCUBA, we sampled 19 sites around San Ambrosio (SA) in February 2013 and 18 sites around Robinson Crusoe and Santa Clara (RC) in January 2014 (Fig 1, S1 Table). Due to their close proximity to one another, Robinson Crusoe and Santa Clara were subsequently grouped and are collectively referred to as RC. At each site, surveys of fishes and benthos were conducted at both 10 and 20 m depth strata for a total of 74 stations. Sites were selected to incorporate representative wave exposures and bottom characteristics. The prevailing wind and swell are from the southwest, resulting in markedly different conditions on the north (sheltered) and south (exposed) shores of both islands.

#### Benthos

At each site we sampled five 10 m-long transects parallel to the shoreline, at both 10 and 20 m depth. For algae, corals, and sessile macro-invertebrates we used a point intercept transect methodology to calculate percent benthic cover, recording the taxa found every 20 cm along a measuring tape. For mobile invertebrates, we counted individuals in five 50 x 50 cm quadrats randomly placed along each of the 10 m transects. Sessile snail worms (Vermetidae), which are more amenable to individual counts, were enumerated by quadrat counts at both locations and also as a percentage of cover at SA. They were excluded from all analyses of benthic cover since they were not included in cover estimates at RC due to logistical constraints.

#### Fishes

At each depth stratum within a site, one diver counted and estimated sizes for all fishes encountered within two (RC) or three (SA) fixed-length (25-m) belt transects whose widths differed depending on direction of swim. Transect bearings were set along isobaths within homogeneous habitats, with each transect separated by at least 5 m. Highly vagile, midwater schooling species (e.g., *Scorpis chilensis*, *Chromis meridian*, *Pseudocaranx chilensis*, *Caprodon longimanus*, *Callanthias platei*) were tallied within an 4-m wide strip surveyed on an initial “swim-out” as the transect line was laid (transect area = 100 m^2^). Small-bodied, less vagile, and more site-attached fishes were tallied within a 2-m wide strip surveyed on the return swim back along the laid transect line (transect area = 50 m^2^). Divers took care to never record individuals entering the field of view from behind in order to avoid counting fishes that tended to follow the divers.

Fishes were identified to species level in all cases [13]. Fish length was estimated to the nearest cm TL. Fishes were tallied by length and individual-specific lengths were converted to body weights. Numerical density (abundance) was expressed as number of individuals per m^2^ and biomass density was expressed as tons per ha. The biomass of individual fishes was estimated using the allometric length-weight conversion: W = aTL^b^, where parameters a and b are species-specific constants, TL is total length in cm, and W is weight in grams. Length-weight fitting parameters were obtained from FishBase [35]. The sum of all individual weights and numerical densities was used to estimate biomass density by species. Fishes were categorized into five trophic groups (piscivores, herbivores, benthic invertivores, benthic invertivores/piscivores, and planktivores) based on published literature. Species that consumed benthic invertebrates and fishes with a trophic number in Fishbase < 4.0 were classified as benthic invertivores/piscivores. Fishes that are consumed directly or used as bait in the lobster and crab fisheries were categorized as resource species (S2 Table).

#### Statistical analysis

Species diversity was calculated using the Shannon-Weaver Diversity Index [36]. Species density per 50 m transect and diversity of sessile benthic organisms were compared using a three-way analysis of variance (ANOVA) with island (RC, SA), exposure (exposed, sheltered), and depth (10 m, 20 m) as fixed factors. Unplanned multiple comparisons were tested using Tukey’s Honestly Significant Difference (HSD) test (α = 0.05). Normality was tested using a Shapiro-Wilks W test (P<0.05) while a Bartlett’s test (P<0.05) was used to examine homogeneity of variance. Numerical density of vermetid snails, the two dominant mobile invertebrates (*Centrostephanus rodgersii* and *Mertensiothuria platei*), and fish assemblage characteristics (e.g., species density, number of individuals, biomass, and resource fish biomass) were all compared in a similar manner. Sessile benthic cover was arcsine-square root transformed, vermetid density were square root transformed, and fish numerical abundance, biomass, and biomass of resource and non-resource species were ln(x+1) transformed prior to conducting the ANOVAs. Densities of the two dominant mobile invertebrates were rank transformed prior to analysis [37].

To describe the pattern of variation in community structure of the sessile benthic organisms and their relationship to environmental gradients, we performed direct gradient analysis (redundancy analysis: RDA) using the ordination program CANOCO version 5.0 [38]. The RDA introduces a series of explanatory (environmental) variables and resembles the model of multivariate multiple regression, allowing us to determine what linear combinations of these environmental variables determine the gradients. Benthic groups that represented > 5% of sessile benthic cover (bare rock, Chlorophyta, erect coralline algae (ECA), *Eisenia cokeri*, Phaeophyceae, Porifera, Rhodophyta, Vermetidae) or > 5% of mobile invertebrate density (*Centrostephanus rodgersii*, *Mertensiothuria platei*, *Parvulastra calcarata*) were included in this analysis. The environmental data matrix included the following variables: island (JF, SA), exposure (exposed, sheltered), and depth (10 m, 20 m). To rank environmental variables in their importance for being associated with the structure of communities, we used a forward selection where the statistical significance of each variable was judged by a Monte-Carlo unrestricted permutation test with 499 permutations [39].

Fish trophic structure was tested for differences between islands using multivariate analysis of variance (MANOVA). Biomass data were 4^th^ root transformed prior to analysis. The multivariate test statistic Pillai’s Trace was used because it is robust to heterogeneity of variance and is less likely to involve type I errors than are comparable tests [40]. We performed univariate ANOVAs when the MANOVA was significant. Similarity of Percentages (SIMPER) was used to determine the living sessile benthic taxa, fish trophic groups, and fish species most responsible for the percentage dissimilarities between islands using Bray-Curtis similarity analysis of hierarchical agglomerative group average clustering [41]. Values in the results are means and one standard deviation of the mean unless otherwise stated.

## Results

### Benthic

#### Sessile benthic cover

Species density of sessile benthic organisms per 50 m transect was higher at RC ($\bar{X}$ = 12.3 ± 3.5) compared to SA ($\bar{X}$ = 8.9 ± 4.2). However there was a significant interaction between island and exposures, with sheltered locations at SA having lower species density compared to the other three island x exposure combinations (Table 1A). Species diversity was significantly higher at RC ($\bar{X}$ = 1.91 ± 0.34) compared to SA ($\bar{X}$ = 1.14 ± 0.57) (Table 1B).

**Table 1:** Comparison of species density and diversity per 50 m transect of sessile benthic cover between islands, exposures, and depth strata.

A. Species density	F	P	Multiple Comparison
Island	2.31	0.024	RC > SA
Exposure	1.81	0.074	
Depth	1.16	0.251	
Island x Exposure	2.45	0.017	RC She = SA Exp = RC Pro > SA She
F_6,73_ = 6.98, p < 0.001			
B. Diversity			
Island	4.53	<0.001	RC > SA
Exposure	1.26	0.211	
Depth	0.89	0.375	
F_6,73_ = 9.93, p < 0.001			

Results of 3-way ANOVA with interactions. Unplanned multiple comparisons were tested using Tukey’s HSD test (α = 0.05). JF–Juan Fernandez, SA–San Ambrosio, She–sheltered, Exp–exposed. Only significant interactions are shown in tables.

**Fig 2:**
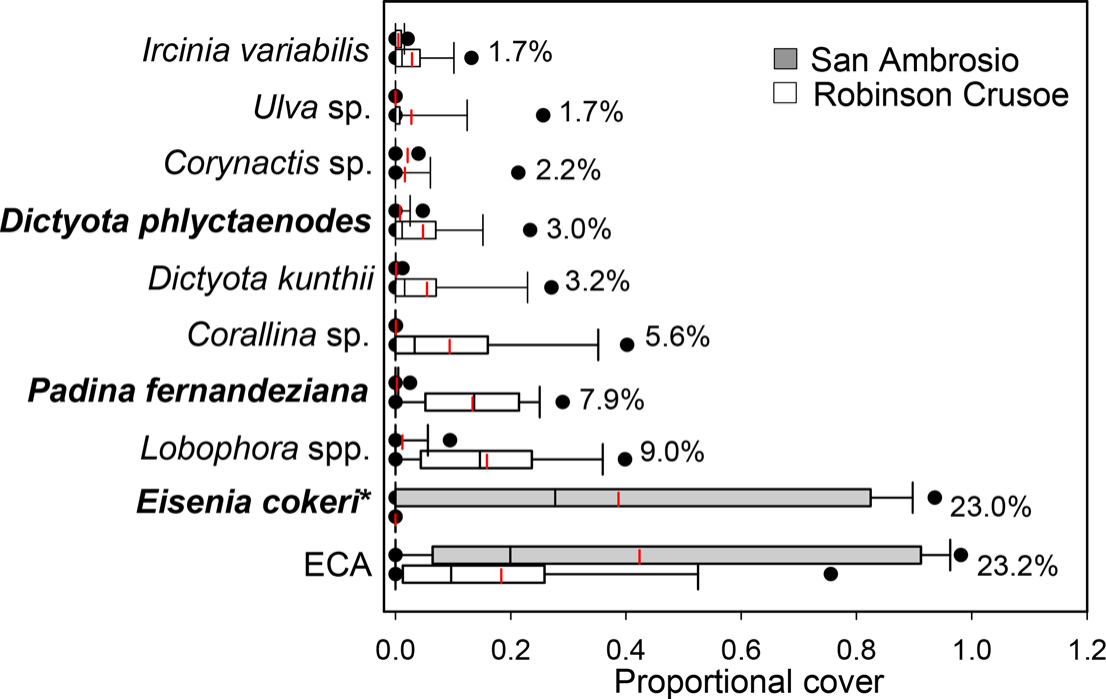
Living sessile taxa most responsible for the dissimilarity between Robinson Crusoe and San Ambrosio islands. Box plots showing median (black line), mean (red dashed line), upper and lower quartiles, and 5th and 95th percentiles for each taxa. Numbers to the right of bars are average dissimilarities based on Similarity of Percentages (SIMPER) analysis. ECA–erect coralline algae. Endemic species are shown in bold. Eisenia cokeri*—Known from Desventuradas Islands and reported from coastal Peru.

Average dissimilarity in living sessile benthic cover between islands (for depth and exposure combined) was 86.8% based on SIMPER analysis (Fig 2). Encrusting coralline algae was the most abundant living sessile benthic taxa at both islands, but cover was 2.3 times higher at SA compared to RC, providing the greatest dissimilarity between islands (23.2%). The kelp *Eisenia cokeri*, which was common on the wave exposed side of SA but was not present at RC, and is only known from Desventuradas Islands in Chile, contributed an additional 23.0% of the dissimilarity between islands. In contrast, RC was dominated by brown algae such as *Lobophora* spp., the endemic *Padina fernandeziana*, and several species of *Dictyota* including the endemic *D*. *phlyctaenodes*.

Worm snails (Vermetidae–*Dendropoma* sp.) were the most abundant taxon on benthic quadrats with 21.1 (± 20.4) individuals m^-2^ at SA and nearly twice that at RC (40.7 ± 33.9 m^-2^) (Table 2A). They were nearly four times more abundant at sheltered sites ($\bar{X}$ = 43.9 ± 29.8) compared to wave exposed areas ($\bar{X}$ = 11.1 ± 13.5). Benthic cover of vermetids at SA was 3.6% (± 4.0), therefore based on the ratio of numerical abundance between the two islands, we estimate benthic cover of vermetids at RC to be ~ 6.9%.

**Table 2:** Comparison of abundance (no. m^-2^) of A. Worm snail (*Dendropoma* sp.), B. Black sea urchin (*Centrostephanus rodgersii*), and C. Sea cucumber (*Mertensiothuria platei*) between islands, exposures, and depth strata.

A. *Dendropoma* sp.	F	P	Multiple Comparison
Island	2.57	0.012	RC > SA
Exposure	6.73	<0.001	She > Exp
Depth	0.59	0.557	
F_6,73_ = 10.3, p < 0.001			
B. *Centrostephanus rodgersii*			
Island	5.46	<0.001	SA > RC
Exposure	4.55	<0.001	She > Exp
Depth	2.23	0.029	Deep > Shallow
Island x exposure	2.56	0.013	SA She > SA Exp = RC Exp = RC She
F_6,73_ = 9.3, p < 0.001			
C. *Mertensiothuria platei*			
Island	0.68	0.501	
Exposure	3.14	0.002	Exp > She
Depth	0.66	0.512	
F_6,73_ = 1.76, p < 0.111			

Results of 3-way ANOVA with interactions. *Dendropoma* sp. was square root transformed, while *C*. *rodgersii* and *M*. *platei* were rank transformed prior to analysis. Unplanned multiple comparisons were tested using Tukey’s HSD test (α = 0.05). JF–Juan Fernandez, SA–San Ambrosio, She–sheltered, Exp–exposed. Only significant interactions are shown in tables.

#### Mobile benthic invertebrates

The sea urchin *Centrostephanus rodgersii* was the most abundant mobile macroinvertebrate at SA (1.5 m^-2^ ± 1.5), accounting for 88% of the total abundance of mobile invertebrates at this island. Its density was five times lower at RC (0.3 m^-2^ ± 0.5), where it only accounted for 29% of mobile macroinvertebrate abundance. Abundance of *C*. *rodgersii* was 77% higher at sheltered (3.59 m^-2^ ± 2.08) compared to exposed sites (2.00 m^-2^ ± 1.91), and similarly higher at deeper (1.17 m^-2^ ± 1.49) relative to shallow sites (0.66 m^-2^ ± 1.07, Table 2B). There was a significant interaction between island and depth, with the sheltered sites at SA having significantly higher densities of *C*. *rodgersii* than the other island x exposure combinations.

The sea cucumber *Mertensiothuria platei*, which is endemic to both island groups, was the most abundant mobile macroinvertebrate at RC, accounting for 46% of the total community (Table 2C). The density of this species was 2.7 times higher at RC compared with SA, where it accounted for 9% of the total macroinvertebrate abundance. It was significantly more abundant at exposed (0.52 m^-2^ ± 1.17) vs. sheltered sites (0.14 m^-2^ ± 0.28), and 4.8 times more abundant at shallow compared to deep sites, although this difference was not statistically significant due to high variability. Extreme values were observed only at the shallow depth stratum, meaning that spatial heterogeneity was restricted to this stratum. *Parvulastra calcarata* is a seastar endemic to the region that accounted for 20% of the macroinvertebrate abundance at RC, where it was five times more abundant than at SA, where it only accounted for 2% of the abundance.

#### Benthic community comparisons

**Fig 3:**
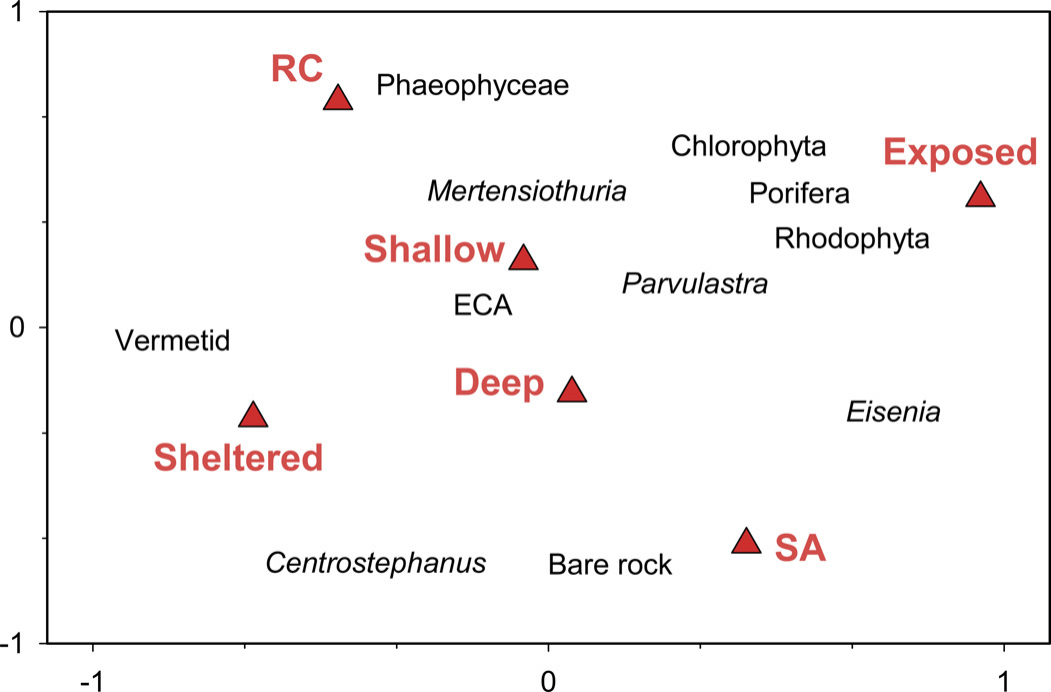
Biplot of results of redundancy analysis on dominant sessile and mobile benthic taxa and environmental data. Data were squareroot transformed and centered prior to analysis. Statistical results are shown in Table 2.

Our data show strong separation in sessile benthic community structure between wave exposure and islands (Fig 3). The first two axes of the RDA biplot explained 43% of the variance in sessile cover and nearly 99% of the benthic-environment relationship (Table 3). The main factors involved in this ordination were wave exposure and island, which were orthogonal to one another in ordination space. Axis 1 separated wave exposures with the most influential response variable scores being *Eisenia cokeri*, which increased towards the exposed areas of SA, and vermetid worms, which increased towards sheltered areas. Axis 2 separated islands with brown algae (Phaeophyceae) in the direction of greater abundance at RC, and bare rock and the sea urchin *Centrostephanus* in the direction of higher prevalence at SA. Exposed areas were characterized by a mix of sponges (Porifera), green algae (Chlorophyta), and red algae (Rhodophyta).

**Table 3:** A. Results of redundancy analysis (RDA) on square root transformed dominant sessile and mobile benthic taxa with environmental variables (e.g., island, wave exposure, and depth). B. Conditional effects of Monte-Carlo permutation results on the redundancy analysis (RDA).

A. Statistic	Axis 1	Axis 2	Axis 3
Eigenvalues	0.30	0.13	0.01
Pseudo-canonical correlation	0.71	0.78	0.34
Explained variation (cumulative)	30.37	43.86	44.42
Explained fitted variation (cumulative)	68.37	98.75	100.00
B. Variable	Pseudo-F	P	% explained
Island	27.5	0.002	27.6
Exposure	15.3	0.003	15.3

### Fishes

#### Fish assemblage characteristics

A total of 30 species of fishes from 21 families were recorded from RC (25 sites) and SA (24 sites, S2). One new record, the kelpfish—*Chironemus delfini*, previously known only from RC was observed at SA during our expedition in the tide pools on the island’s west side.

**Fig 4:**
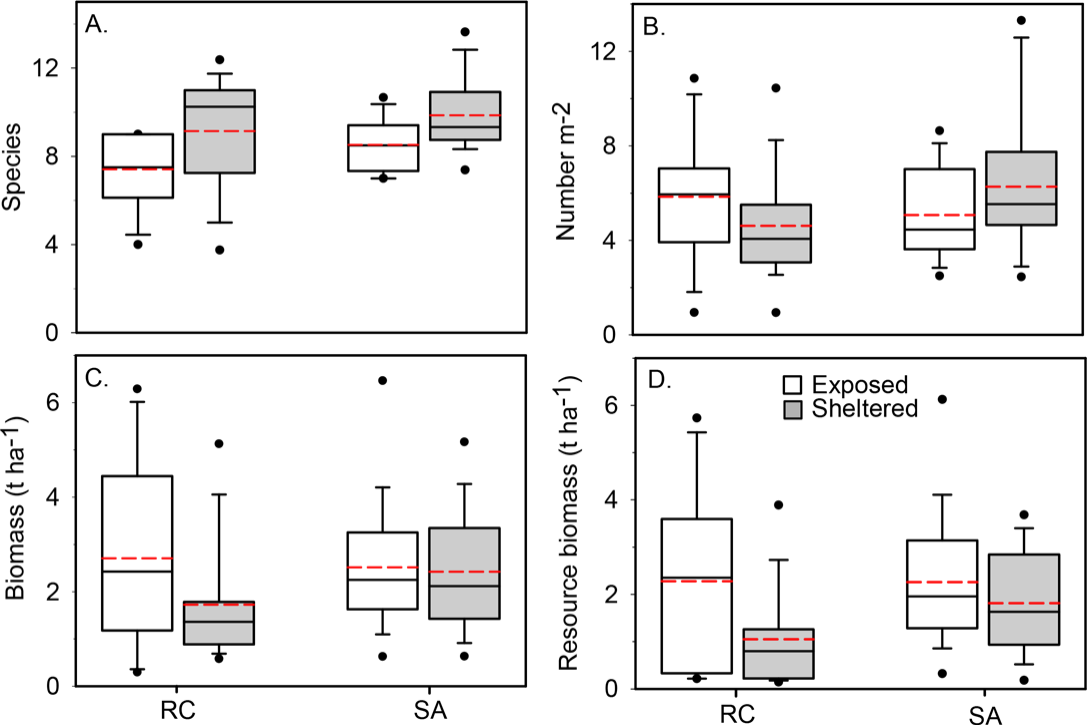
Comparisons of fish assemblage structure between Robinson Crusoe (RC) and San Ambroio SA. Box plots showing median (black line), mean (red dashed line), upper and lower quartiles, and 5th and 95th percentiles for each assemblage characteristic. (A) Species density (number of species per transect), (B) Number of individuals (number m^-2^), (C) Biomass (t ha^-1^), and (D) Resource fish biomass (t ha^-1^). See Table 4 for statistical results.

Average fish species density on transects was low overall ($\bar{x}$ = 8.9 ± 2.0), but significantly higher at SA compared with RC, higher at sheltered vs. exposed sites, and higher at deeper vs. shallow sites (Table 4, Fig 4). Numerical abundance averaged 5.4 individuals m^-2^ (± 2.5) overall and showed no difference among any factor. Biomass averaged 2.3 (± 1.4) t ha^-1^ and was not different among major factors although the interaction of exposure and depth was significant, with deep sheltered sites having significantly higher biomass than shallow sheltered sites.

Resource fish biomass was significantly greater at SA compared to RC by 39% and significantly greater at exposed vs. sheltered sites by 62% (Table 4, Fig 4). Within RC, resource fish biomass was > 2 times greater at the exposed, remote sites compared to the sheltered sites closer to the harbor (F_1,35_ = 2.33, p = 0.026). There was no significant difference in resource fish biomass between depth strata surveyed around RC (F_1,35_ = 0.82, p = 0.42). Non-resource fishes showed the opposite response, with 40% higher biomass at RC compared to SA (F_1,67_ = 2.79, p = 0.007), and 55% higher biomass in sheltered vs. exposed locations (F_1,67_ = 3.75, p < 0.001).

**Table 4:** Comparison of fish assemblage metrics between islands, exposures, and depth strata.

A. Species density	F	P	Multiple Comparison
Is	2.16	0.035	SA > RC
Exposure	3.64	<0.001	She > Exp
Depth	2.59	0.011	Deep > Shallow
B. Numerical abundance (no. m^-2^)			
Is	1.00	0.322	
Exposure	0.23	0.818	
Depth	0.46	0.649	
C. Biomass (t ha^-1^)			
Is	1.65	0.104	
Exposure	1.27	0.210	
Depth	1.43	0.157	
Exp. X depth	2.12	0.038	She.20 Exp.10 Exp.20 She.10 ^______________________________^
D. Resource biomass (t ha^-1^)			
Is	2.01	0.049	SA > RC
Exposure	2.57	0.012	Exp > She
Depth	1.64	0.106	

Results of 3-way ANOVA with interactions. Numerical abundance, total biomass, and resource biomass were ln(x+1) transformed prior to statistical analyses. Unplanned multiple comparisons were tested using Tukey’s HSD test (α = 0.05). JF–Juan Fernandez, SA–San Ambrosio, She–sheltered, Exp–exposed. Only significant interactions are shown in tables. Underlined factors are not significantly different (α = 0.05).

#### Biodiversity and endemism

There was a total number of 25 species of fishes recorded on transects at SA and 24 at RC (Table 5). The number of endemic species was extremely high with 87.5% of the species at RC and 72% at SA consisting of regional (Juan Fernandez and Desventuradas) endemics. Endemics accounted for nearly 99% of the numerical abundance of all fishes observed on transects at RC and 96% at SA. Nearly 88% of fish biomass at RC and 75% at SA consisted of these regional endemics.

**Table 5:** Affinities of fish species observed on quantitative transects around Robinson Crusoe (RC) and San Ambrosio (SA) islands.

	Species observed on transects		Numerical abundance (number m^-2^)	
**Affinity**	RC	SA	RC	SA
**Desventuradas & Juan Fernandez**	21	18	5.01 (2.41)	4.57 (1.85)
**Desventuradas only**		1		1.11 (1.39)
**Juan Fernandez only**	1		0.01 (0.04)	
**Circumtropical**	1	1	<0.01 (0.10)	0.02 (0.04)
**South Pacific**	1	2	0.01 (0.01)	0.01 (0.1)
**Desventuradas & Easter Island**		1		<0.01 (0.01)
**Indo-Pacific**		1		<0.01 (<0.1)
**Coastal Chile**		1		<0.01 (<0.1)
**Total**	24	25		

Values in parentheses are SD. Species ordered by numerical abundance.

#### Fish trophic structure

**Fig 5:**
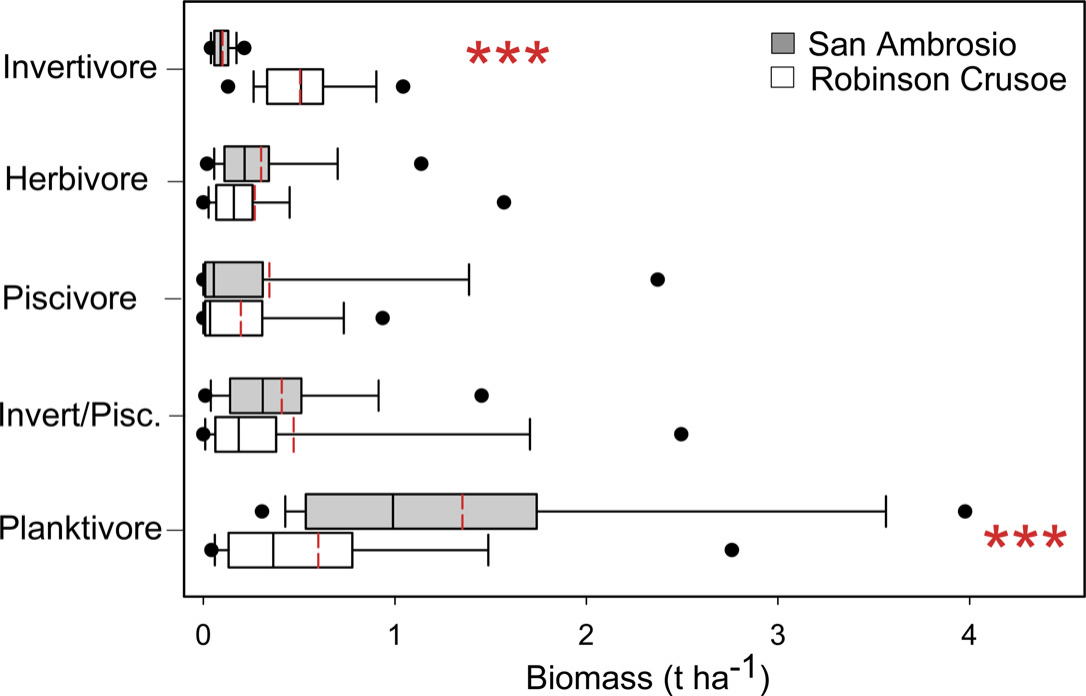
Comparisons of fish trophic groups between Robinson Crusoe and San Ambrosio islands. Box plots showing median (black line), mean (red dashed line), upper and lower quartiles, and 5^th^ and 95^th^ percentiles. Values are biomass (t ha^-1^). Invert/Pisc.–invertivores/piscivores. Statistical results of 1-way ANOVAs testing island differences. Trophic groups with *** are significantly different at p < 0.001.

Fish trophic structure was significantly different between SA and RC (Pillai’s Trace, F_4,69_ = 36.4, p < 0.001). Univariate ANOVAs were performed on each trophic group following the significant results of the MANOVA. Planktivore biomass was 2.3 times higher at SA compared to RC (F_1,74_ = 22.6, p < 0.001) and accounted for nearly 40% of the dissimilarity between islands (SIMPER Analysis). This trophic group comprised 54% of total biomass at SA but only 29% at RC (Fig 5). Biomass of benthic invertivores was significantly different between islands (F_1,74_ = 132.4, p < 0.001) and this trophic group combined with benthic invertivores/piscivores together accounted for 47% of the biomass at RC but only 20% at SA.

#### Fish species composition

Fish species composition between SA and RC were distinct (average dissimilarity for biomass = 65.6%, SIMPER Analysis). The Chilean sweeper (*Scorpis chilensis*) was the most important species by weight at both locations, accounting for 32% of total fish biomass at SA and 24% at RC, although absolute biomass was 63% higher at SA, which accounted for 22% of the dissimilarity between assemblages (Table 6). The Juan Fernandez trevally (*Pseudocaranx chilensis*) was the next most important species by weight at both locations, accounting for an additional 21% of the total biomass at RC and 14% at SA. This species had 23% greater biomass at RC compared to SA and contributed 14% to the dissimilarity between these assemblages. Ranking third in overall weight at SA was the yellowtail Amberjack (*Seriola lalandi*), which comprised an additional 13% of total biomass around this island. Biomass of this highly prized species was 2.7 times higher at SA compared to RC, where it comprised 6% of the total biomass, ranking sixth for the island. Two small labrids, the reticulated wrasse (*Malapterus reticulatus*), and Gay’s wrasse (*Pseudolabrus gayi*) together accounted for 23% of the total biomass at RC where they ranked third and fourth in total weight, respectively.

**Table 6:** Fish species most responsible for the dissimilarity between Robinson Crusoe (RC) and San Ambrosio (SA) islands based on Similarity of Percentages (SIMPER) analysis.

	Biomass—t ha^-1^ (% total)		Av. Diss	Contrib. %
Species	SA	RC	(± sd)	
*Scorpis chilensis*	0.80 (31.9)	0.49 (24.1)	14.5 (1.6)	22.13
*Pseudocaranx chiliensis*	0.34 (13.6)	0.42 (20.6)	9.0 (0.1)	13.66
*Seriola lalandi*	0.33 (13.2)	0.12 (5.9)	7.3 (0.8)	11.12
*Girella albostriata*	0.25 (10.0)	0.17 (8.3)	6.2 (1.0)	9.48
*Malapterus reticulatus*	0.01 (0.4)	0.24 (11.8)	5.7 (1.6)	8.67
*Caprodon longimanus*	0.23 (9.2)	0.09 (4.4)	5.4 (0.8)	8.21
*Chromis meridiana*	0.23 (9.2)	-	5.0 (1.1)	7.69
*Pseudolabrus gayi*	0.06 (2.4)	0.23 (11.3)	4.1 (1.2)	6.29
*Callanthias platei*	0.08 (3.2)	-	1.7 (0.6)	2.53
*Gymnothorax porphyreus*	0.06 (2.4)	0.04 (2.0)	1.5 (0.8	2.27

Values for islands are biomass (t ha^-1^) with percentage of total in parentheses. Avg. Diss.–average dissimilarity with standard deviation in parentheses.

### Discussion

The Desventuradas and Juan Fernández islands possess unique marine ecosystems that consist of a mix of tropical, subtropical, and temperate species. The major difference observed in the benthic communities between San Ambrosio (SA) in Desventuradas and Robinson Crusoe (RC) in Juan Fernández is the presence of the kelp *Eisenia cokeri* at SA, where it forms dense beds at exposed areas of the island. This species is only known from Desventuradas Islands (with a limited distribution along the coast of Perú), and constitutes some of the lowest latitude insular kelp communities in the entire Pacific [42–43]. This single species creates a unique habitat that provides refuges, feeding, and nursery grounds for a wide range of benthic species, and also appears to be a critical nursery habitat for several pelagic species including the endemic Juan Fernández jack (*Pseudocaranx chilensis*) (Pers. Obs.). This fish species is an important food fish and bait resource for Juan Fernández fishermen but no subadults were observed during our surveys at RC. This lack of subadult jacks around RC may mean that: (1) recruitment is very episodic, as is common in many isolated locations; or (2) recruitment of jacks occurs in a habitat that we did not encounter. These hypotheses are not mutually exclusive and further research will be required to answer this question.

The benthic community at RC was more diverse compared to SA and this may partially be explained by the larger area of the island, more diverse habitats, and greater protection from large waves. At RC island, however, there is a complete absence of the kelp *Eisenia cokeri*. The dominance of sea urchin barrens at sheltered sites around SA was surprising since this habitat is usually associated with the loss of keystone species and trophic cascades in other areas of the world [44–45]. Urchin barrens may be formed during episodic population explosions of these organisms, as has been reported from other isolated islands in the Pacific [46]. Given the nearly pristine condition at SA and the numerous lobsters observed on our submersible surveys, these urchin barrens may represent a natural state and may contribute to the lower benthic diversity observed at SA compared to RC.

Lord Howe Island in the southwest Pacific is also an isolated, endemism hotspot with a number of similarities to Desventuradas and Juan Fernández [47–48]. Lord Howe possesses a mix of tropical, subtropical, and temperate species owing to its location at the boundary between the Coral and Tasman seas, with distinctive coral reef and macroalgal communities that are strongly influenced by wave exposure [49]. Barrens of the sea urchin *C*. *rodgersii* have also been reported from Lord Howe [49], and it has been suggested that formation of these barrens can be facilitated by increases in water temperature, which can stress native communities and contribute to larval dispersal [50–51]. Increases in ocean temperatures around Desventuradas could affect the resilience of the *Eisenia* kelp beds, making them more susceptible to disturbance and predation by sea urchins, as has been shown elsewhere [52].

Although these islands are close to mainland Chile (~500 mi), the cold, nutrient-rich waters of the Humboldt Current act as an effective barrier that separates the marine life found at these islands from the South American coast. The most abundant mobile macroinvertebrate in our surveys was *Centrostephanus rodgersii*, whose distribution includes eastern Australia and New Zealand, but not coastal South America [53]. Similarly, fishes found at these islands have a great affinity with the central Pacific than coastal Chile [13–14]. The juxtaposition of these tropical and sub-tropical species with kelp beds and fur seals, which are more typical of temperate ecosystems, creates a very distinctive ecological setting.

Endemics comprised 72% of the total number of fish species observed on transects at SA and 87.5% of the species at RC. More remarkable is the fact that nearly 99% of the numerical abundance of all fishes observed on transects at RC and 96% at SA are endemic to these islands only. The total number of coastal fish species known from the Desventuradas and Juan Fernández islands is 52, of which 32 (61.5%) are endemic [13]. These values are two to four times higher than those reported from other locations (e.g., Hawaii– 25%, [54], Easter Island– 21.7%, [55], Marquesas– 13.7%, [22], and highlight the global significance and uniqueness of these biodiversity hotspots. The Desventuradas and Juan Fernandez islands have extremely high levels of endemism, matched only by other isolated biogeographic provinces (e.g., Tropical Eastern Pacific, Antarctic [17, 56]). However the much smaller geographic range of these islands makes them strikingly unique globally.

The near absolute dominance of endemics in the fish assemblages at SA and RC is without precedent, and is possibly explained by their extreme geographic and oceanographic isolation. Endemic reef fishes are often associated with isolated islands, where they may have evolved traits that generally are associated with high local abundance [57–59]. The high abundance of marine endemic species may buffer them from low genetic diversity and stochastic processes, such as high recruitment and climatological variability [59]. Despite their high adaptive capacity, most of the recorded extinctions in the marine environment have been those species with small range sizes [60]. A recent analysis of global reef fish assemblages found that, among all locations examined, functional sensitivity (i.e. the proportion of functional groups with only a single species present) was highest in the Juan Fernandez Archipelago [61], thus emphasizing the vulnerability of these islands.

The marine flora of the Juan Fernández Islands exhibits high endemism (~30%), but also a number of species with peculiar affinities that include the southern tip of South America, Southern Australia, New Zealand, and several sub-Antarctic islands [62]. Some common taxa such as certain brown algal taxa reported here as *Lobophora* spp. are still undescribed, despite their prevalence in the ecosystem [63]. Despite the relatively close proximity of the islands, the marine flora of Easter Island is more similar to the central and western Pacific than to Juan Fernández. This is consistent with predictions of limited species exchange across the northward flow of the Chile-Perú current system [62, 64–65], therefore accounting for the isolation and high levels of endemism but also connectivity with distant locations. Similarly, the marine flora of Desventuradas has a strong affinity with the Juan Fernández Islands and limited overlap with species found on the continent [66–67]. In addition to the marine flora, the endemic sea cucumber, *Mertensiothuria platei*, and sea star, *Parvulastra calcarata*, were important components of the benthos, comprising > 66% of the mobile macroinvertebrate abundance at RC.

Fish biomass at both locations was large compared to many locations throughout the Pacific [68–69]. However, resource fish biomass was 39% higher around SA compared to RC, and higher at exposed sites at RC compared to sheltered sites closer to the port, which possibly suggest the effects of fishing (for bait and human consumption) around RC. Although geography (productivity, temperature) and the presence of 120,000 fur seals at RC may contribute to these observed differences, the contrasts in biomass of resource and non-resource fishes between islands and around RC likely indicates selective fishing. In fact, some of the highest fish biomass observed around Robinson Crusoe and Santa Catalina islands were adjacent to the largest seal colonies on these islands [70]. More than 90% of the diet of the Juan Fernández fur seal consists of cephalopods, along with midwater (Myctophidae) and epipelagic (Scomberesocidae) fishes [71], so seals likely have minimal impact on the nearshore fish fauna. Despite recent advances towards a sustainable lobster fishery within the Juan Fernández Archipelago, a diverse assemblage of fishes are increasingly caught as bait for this fishery [27, 30], emphasizing the need for broader resource management.

### Conclusions

This distinctive mix of tropical, sub-tropical, and temperate species makes these islands extremely unique. The levels of endemism in the fish assemblages are unprecedented and the benthic community is dominated by species that are either endemic, or possess distribution patterns and affinities that are novel for this region. These islands share a number of unique ecological features but different levels of human influence, and therefore offer us a valuable perspective on how remote oceanic marine ecosystems function and how best to manage them. On October 5, 2015, the Chilean Government announced the creation of the Nazca-Desventuradas Marine Park, which encompasses ~ 297,518 km^2^ around San Ambrosio and San Félix islands, making it the largest fully protected marine reserve in the Americas. In 2014, Chile created the Juan Fernández Multi-Use Marine Protected Area, covering over 12,000 km^2^ of the archipelago. This study highlights the uniqueness of the marine life at Juan Fernández and Desventuradas islands and the new management protection will greatly aid in the conservation of these globally important endemic hotspots

## Supporting Information

S1 Table Locations surveys during expeditions to San Ambrosio Island in the Desventuradas islands in February 2013 and Robinson Crusoe and Santa Clara islands in the Juan Fernández islands in January 2014. (DOCX)

S2 Table Fishes observed around San Ambrosio Island and Robinson Crusoe and Santa Clara islands. Resource species are those consumed directly or used as bait in the lobster and crab fisheries.
(DOCX)

## References

[1] Medina JT. El Piloto Juan Fernández, descubridor de las islas que Ilevan su nombre, y Juan Jufreé, armador de la expedicioón que hizo en busca de otras en el Mar del Sur Estudio histórico. Santiago de Chile: Imprenta elzeviriana; 1918.

[2] Bahamonde N. San Félix y San Ambrosio, las islas llamadas Desventuradas In: Castilla JC, editor. Islas Oceánicas Chilenas: Conocimiento científico y necesidades de investigaciones. Santiago: Universidad Católica de Chile; 1987 pp. 85–100.

[3] Castilla JC. Islas océanicas Chilenas: conociemento cientifi co y necesidades de investigaciones Santiago: Universidad Católica de Chile; 1987.

[4] Fernández M, Hormazábal S. Overview of recent advances in oceanographic, ecological and fisheries research on oceanic islands in the southeastern Pacific Ocean. Lat Am J Aquat Res. 2014; 42: 666–672.

[5] Rodríguez-Ruiz MC, Andreu-Cazenave M, Ruz CS, Ruano-Chamorro C, Ramírez F, González C, et al Initial assessment of coastal benthic communities in the Marine Parks at Robinson Crusoe Island. Lat Am J Aquat Res. (2014; 42: 918–936.

[6] Pérez-Matus A, Ramírez F, Eddy TD, Cole R. Subtidal reef fish and macrobenthic community structure at the temperate Juan Fernandez Archipelago, Chile. Lat Am J Aquat Res. 2014; 42: 814–826.

[7] Ramírez F, Pérez-Matus A, Eddy TD, Landaeta MF. Trophic ecology of abundant reef fish in a remote oceanic island: coupling diet and feeding morphology at the Juan Fernandez Archipelago, Chile. J Mar Biol Assoc UK. 2013; 93: 1457–1469.

[8] Tulke H. La Isla de Juan Fernández y sus problemas. Scientia. 1954; 21: 140–165.

[9] Castilla JC, Oliva D. Islas Oceánicas Chilenas: Aspectos descriptivos y potencialidades In: Castilla JC, editor. Islas Oceánicas chilenas. Conocimiento Científico y necesidades de investigación. Santiago: Universidad Católica de Chile; 1987 pp. 15–36.

[10] Anderson AJ, Haberle S, Rojas G, Seelenfreund A, Smith I, Worthy T. An archaeological exploration of Robinson Crusoe Island, Juan Fernandez Archipelago, Chile In: Holdaway S, Brown D, editors. Fifty years in the field: essays in honour and celebration of Richard Shutler Jr’s archaeological career. Auckland: New Zealand Archaeological Association; 2002 pp 239–249.

[11] Sepúlveda JI. Peces de las islas oceánicas chilenas In: Castilla JC, editor. Islas Oceánicas chilenas. Conocimiento Científico y necesidades de investigación Santiago: Universidad Católica de Chile; 1987 pp. 225–245.

[12] Haberle S Juan Fernández Islands In: Gillespie RG, Clague DA, editors. Encyclopedia of Islands. Berkeley: University of California Press; 2009 pp 507–509.

[13] Dyer BS, Westneat MW. Taxonomy and biogeography of the coastal fishes of Juan Fernández Archipelago and Desventuradas Islands, Chile. Rev Biol Mar Oceanogr. 2010; 45: 589–617.

[14] Pequeño G, Sáez S. Los peces litorales del archipiélago de Juan Fernández (Chile): endemismo y relaciones ictiogeográficas. Investig Mar. 2000; 28: 27–37. 10.4067/S0717-71782000002800004

[15] Briggs JC, Bowen BW. A realignment of marine biogeographic provinces with particular reference to fish distributions. J Biogeogr. 2012; 39: 12–30.

[16] Pequeño G, Lamilla J. The littoral fish assemblage of the Desventuradas Islands (Chile) has zoogeographical affinities with the Western Pacific. Global Ecol Biogeogr. 2000; 9: 431–437.

[17] Robertson DR, Cramer KL. Shore fishes and biogeographic subdivisions of the Tropical Eastern Pacific. Mar Ecol Prog Ser. 2009; 380: 1–17.

[18] Moritz C. Strategies to protect biological diversity and the evolutionary processes that sustain it. Syst Biol. 2002; 51: 238–254. 12028731 10.1080/10635150252899752

[19] Bowen BW, Rocha LA, Toonen RJ, Karl SA. The origins of tropical marine biodiversity.Trends Ecol Evolut 2013; 28: 359–366. 10.1016/j.tree.2013.01.018 23453048

[20] Brooks TM, Mittermeier RA, da Fonseca GAB, Gerlach J, Hoffmann M, Lamoreux JF, et al Global biodiversity conservation priorities. Science. 2006; 313: 58–61. 16825561 10.1126/science.1127609

[21] Roberts CM, McClean CJ, Veron JE, Hawkins JP, Allen GR, McAllister DE, et al Marine biodiversity hotspots and conservation priorities for tropical reefs. Science. 2002; 295: 1280–1284. 11847338 10.1126/science.1067728

[22] Delrieu-Trottin E, Williams JT, Bacchet P, Kulbicki M, Mourier J, Galzin R, et al Shore fishes of the Marquesas Islands, an updated checklist with new records and new percentage of endemic species. Check List. 2002; 11: 1758.

[23] Croxall JP, Butchart SH, Lascelles B, Stattersfield AJ, Sullivan B, Symes A, Taylor P. Seabird conservation status, threats and priority actions: a global assessment. Bird Conserv Int. 2012; 22: 1–34.

[24] BirdLife International. Species factsheet: *Pterodroma defilippiana*; 2015. Database: www.birdlife.org. Accessed 11 June 2015.

[25] Martínez C, Boré D. Catálogo de recursos pesqueros Chile CORFO-IFOP; 1980, 92 pp.

[26] Castilla JC, Defeo O. Latin American benthic shellfisheries: emphasis on co-management and experimental practices. Rev Fish Biol Fish. 2001; 11: 1–30.

[27] Eddy TD, Gardner JP, Pérez-Matus A. Applying fishers' ecological knowledge to construct past and future lobster stocks in the Juan Fernández Archipelago, Chile. PLoS ONE. 2010; 5(11): e13670 10.1371/journal.pone.0013670 21079761 PMC2974625

[28] Ernst B, Manríquez P, Orensanz JM, Roa R, Chamorro J, Parada C. Strengthening of a traditional territorial tenure system through protagonism in monitoring activities by lobster fishermen from the Juan Fernández Islands, Chile. Bull Mar Sci. 2010; 86: 315–338.

[29] Arana P. Perspectivas históricas y proyecciones de la actividad pesquera realizada en el archipiélago de Juan Fernández, Chile In: Castilla JC, editor. Islas Oceánicas Chilenas: Conocimiento Científico y Necesidades de Investigaciones. Santiago: Universidad Católica de Chile; 1987 pp 319–353.

[30] Ernst B, Chamorro J, Manríquez P, Orensanz JL, Parma AM, Porobic J., Román C. Sustainability of the Juan Fernández lobster fishery (Chile) and the perils of generic science-based prescriptions. Glob Environ Change. 2013; 23: 1381–1392.

[31] González-Ferrán O. Evolución geológica de las islas chilenas en el océano Pacífico In: Castilla JC, editor. Islas oceánicas chilenas: conocimiento científico y necesidades de investigación. Santiago: Universidad Católica de Chile; 1987 pp. 37–54.

[32] Rodrigo C, Lara L. Plate tectonics and the origin of the Juan Fernández Ridge: analysis of bathymetry and magnetic patterns. Lat Am J Aquat Res. 2014; 42: 907–917.

[33] Rodrigo C, Díaz J, González-Fernández A. Origin of the Easter Submarine Alignment: morphology and structural lineaments. Lat Am J Aquat Res. 2014; 42: 857–870.

[34] NOAA NMFS SWFSC ERD. SST, POES AVHRR, GAC, Global, Day and Night (Monthly Composite). Available: http://coastwatch.pfeg.noaa.gov/erddap/info/erdAGsstamday/index.html

[35] Froese R, Pauly D. FishBase; 2011. World Wide Web electronic publication. www.fishbase.org. Accessed 11 June 2015.

[36] Ludwig JA, Reynolds JF. Statistical Ecology. Hoboken, New York: John Wiley & Sons; 1988.

[37] Rayner JCW, Best DJ. Extended ANOVA and rank transform procedures. Aust NZ J Stat. 2013; 55: 305–319.

[38] ter Braak CJF, Šmilauer P. (2012) Canoco Reference Manual and User’s Guide: Software for Ordination, Version 5.0. Microcomputer Power: Ithaca, NY, USA.

[39] ter Braak CJ, Verdonschot PF. Canonical correspondence analysis and related multivariate methods in aquatic ecology. Aquat Sci. 1995; 57: 255–289.

[40] Green RH. Sampling Design and Statistical Methods for Environmental Biologists. New York: Wiley Interscience; 1979.

[41] Clarke KR. Non-parametric multivariate analyses of changes in community structure. Aust J Ecol. 1993; 18: 117–143.

[42] Etcheverry H. Algas Marinas de las Islas Oceánicas Chilenas. Rev Biol Mar Oceanogr. 1960; 10: 83–132.

[43] Ramírez ME, Santelices B. Catálogo de las algas marinas bentónicas de la costa del Pacífico temperado de Sudamérica Monografías Biológicas, 5 Santiago: Pontificia Universidad Católica de Chile 1991.

[44] Tegner MJ, Dayton PK. Ecosystem effects of fishing in kelp forest communities. ICES J Mar Sci. 2000; 57: 579–589.

[45] Valentine JP, Edgar GJ. Impacts of a population outbreak of the urchin *Tripneustes gratilla* amongst Lord Howe Island coral communities. Coral Reefs. 2010; 29: 399–410.

[46] Ling SD, Scheibling RE, Rassweiler A, Johnson CR, Shears N, Connell SD. et al Global regime shift dynamics of catastrophic sea urchin overgrazing. Philos Trans R Soc Lond B Biol Sci. 2015; 370: 20130269 10.1098/rstb.2013.0269

[47] Francis MP. Checklist of the coastal fishes of Lord Howe, Norfolk, and Kermadec Islands, southwest Pacific Ocean. Pac Sci. 1993; 47: 136–170.

[48] Lindsay MJ, Patterson HM, Swearer SE. Habitat as a surrogate measure of reef fish diversity in the zoning of the Lord Howe Island Marine Park, Australia. Mar Ecol Prog Ser. 2008; 353: 265–273.

[49] Edgar GJ, Davey A, Kelly G, Mawbey RB, Parsons K. Biogeographical and ecological context for managing threats to coral and rocky reef communities in the Lord Howe Island Marine Park, south‐western Pacific. Aquat Conserv. 2010; 20: 378–396.

[50] Pederson HG, Johnson CR. Predation of the sea urchin Heliocidaris erythrogramma by rock lobsters (*Jasus edwardsii*) in no-take marine reserves. J Exp Mar Biol Ecol. 2006 336: 120–134.

[51] Hoegh-Guldberg O, Bruno JF. The impact of climate change on the world’s marine ecosystems. Science. 2010; 328: 1523–1528. 10.1126/science.1189930 20558709

[52] Wernberg T, Thomsen MS, Tuya F, Kendrick GA, Staehr PA, Toohey BD. Decreasing resilience of kelp beds along a latitudinal temperature gradient: potential implications for a warmer future. Ecol Lett. 2010; 13: 685–694. 10.1111/j.1461-0248.2010.01466.x 20412279

[53] Mortensen T. A Monograph of the Echinoidea. III, 1. Aulodonta, with additions to Vol. II (Lepidocentroida and Stirodonta). Copenhagen: CA Reitzel; 1940.

[54] Randall JE. Reef and shore fishes of the Hawaiian Islands Honolulu Sea Grant College Program, University of Hawaiʻi; 2007.

[55] Randall JE, Cea A. Shore fishes of Easter Island Honolulu University of Hawaiʻi Press; 2011.

[56] Briggs JC. Marine centres of origin as evolutionary engines. J Biogeogr. 2003; 30:1–18.

[57] Randall JE. The endemic shore fishes of the Hawaiian Islands, Lord Howe Island and Easter Island. Colloque Commerson 1973 O.R.S.T.O.M. Travauxet Documents No. 47: 49–73.

[58] Jones GP, Caley MJ, Munday PL. Rarity in coral reef fish communities In: Sale PF, editor. Coral Reef Fishes: Dynamics and Diversity in a Complex Ecosystem. San Diego, California: Academic Press, 2002 pp. 81–101.

[59] Hobbs J-PA, Jones GP, Munday PL. Extinction risk in endemic marine fishes. Conserv Biol. 2011; 25: 1053–1055. 10.1111/j.1523-1739.2011.01698.x 21676030

[60] Dulvy NK, Sadovy Y, Reynolds JD. Extinction vulnerability in marine populations. Fish Fish. 2003; 9: 261–285.

[61] Parravicini V, Villéger S, McClanahan T, Arias Gonzalez J, Bellwood D, Belmaker J, et al Global mismatch between species richness and vulnerability of reef fish assemblages. Ecol Lett. 2014; 17: 1101–1110. 10.1111/ele.12316 24985880

[62] Santelices B, Meneses I. A reassessment of the phytogeographic characterization of temperate Pacific South America. Rev Chil Hist Nat. 2000; 73: 605–614.

[63] Vieira C, D'hondt S, De Clerck O, Payri CE. Toward an inordinate fondness for stars, beetles and Lobophora? Species diversity of the genus Lobophora (Dictyotales, Phaeophyceae) in New Caledonia. J Phycol. 2014; 50: 1101–1119. 26988791 10.1111/jpy.12243

[64] Tellier F, Meynard AP, Correa JA, Faugeron S, Valero M. Phylogeographic analyses of the 30° S south-east Pacific biogeographic transition zone establish the occurrence of a sharp genetic discontinuity in the kelp *Lessonia nigrescens*: Vicariance or parapatry? Mol Phylogenet Evol. 2009; 53: 679–693. 10.1016/j.ympev.2009.07.030 19647087

[65] Miloslavich P, Klein E, Díaz JM, Hernandez CE, Bigatti G, Campos L, et al Marine biodiversity in the Atlantic and Pacific coasts of South America: knowledge and gaps. PloS one. 2001; 6: e14631. 10.1371/journal.pone.0014631 PMC3031619 21304960

[66] Meneses I, Hoffmann AJ. Contribution to the marine algal flora of San Felix Island, Desventuradas Archipelago, Chile. Pac Sci. 1994; 48: 464–474.

[67] Silva PC, Chacana ME. Marine algae from Islas San Félix y San Ambrosio (Chilean Oceanic Islands). Cryptogamie, Algologie. 2005; 26: 103–118.

[68] Sandin SA, Smith JE, DeMartini EE, Dinsdale EA, Donner SD, et al Degradation of coral reef communities across a gradient of human disturbance. PLoS ONE 2008; 3(2):e1548 10.1371/journal.pone.0001548 18301734 PMC2244711

[69] Williams ID, Richards BL, Sandin SA, Baum JK, Schroeder RE, Nadon MO, et al Differences in reef fish assemblages between populated and remote reefs spanning multiple archipelagos across the central and western Pacific. J Mar Biol. 2010; 2011: 1–14. 10.1155/2011/826234

[70] Osman LP. Population status, distribution and foraging ecology of *Arctocephalus philippii* (Peters 1866) at Juan Fernandez Archipelago. Ph.D. dissertation, Universidad Austral de Chile, Valdivia, Chile. 2008.

[71] Acuña HO, Francis JM. Spring and summer prey of the Juan Fernandez fur seal, *Arctocephalus philippii*. Can J Zool. 1995; 73: 1444–1452.

