# Supplementary material for: Marine Biodiversity in Juan Fernández and Desventuradas Islands, Chile: Global Endemism Hotspots
Source: PLoS One. 2016 Jan 6;11(1):e0145059. doi: 10.1371/journal.pone.0145059 (PMC4703205; doi:10.1371/journal.pone.0145059)
Supplement: S1 Table — (DOCX) [file pone.0145059.s001.docx]

**S1Table.**

Locations surveys during expeditions to San Ambrosio Island in the Desventuradas islands in February 2013 and Robinson Crusoe and Santa Clara islands in the Juan Fernández islands in January 2014.

| Archipelago | Island | Date | Station | Exposure | Latitude | Longitude |
| --- | --- | --- | --- | --- | --- | --- |
| Desventuradas | San Ambrosio | 15-Feb-13 | M1 | Exposed | -26.3443 | -79.8618 |
| Desventuradas | San Ambrosio | 15-Feb-13 | M10 | Protected | -26.3442 | -79.9051 |
| Desventuradas | San Ambrosio | 13-Feb-13 | M11 | Protected | -26.3388 | -79.9026 |
| Desventuradas | San Ambrosio | 21-Feb-13 | M11A | Protected | -26.3414 | -79.9044 |
| Desventuradas | San Ambrosio | 13-Feb-13 | M12 | Protected | -26.3384 | -79.8996 |
| Desventuradas | San Ambrosio | 20-Feb-13 | M13 | Protected | -26.3373 | -79.8938 |
| Desventuradas | San Ambrosio | 14-Feb-13 | M14 | Protected | -26.3362 | -79.8885 |
| Desventuradas | San Ambrosio | 22-Feb-13 | M14A | Protected | -26.3371 | -79.8804 |
| Desventuradas | San Ambrosio | 14-Feb-13 | M15 | Protected | -26.3361 | -79.8846 |
| Desventuradas | San Ambrosio | 14-Feb-13 | M16 | Protected | -26.3375 | -79.8777 |
| Desventuradas | San Ambrosio | 13-Feb-13 | M17 | Protected | -26.3399 | -79.8728 |
| Desventuradas | San Ambrosio | 21-Feb-13 | M18 | Exposed | -26.3423 | -79.8693 |
| Desventuradas | San Ambrosio | 22-Feb-13 | M2 | Exposed | -26.3455 | -79.8734 |
| Desventuradas | San Ambrosio | 10-Feb-13 | M3 | Exposed | -26.3481 | -79.8783 |
| Desventuradas | San Ambrosio | 15-Feb-13 | M4 | Exposed | -26.3495 | -79.882 |
| Desventuradas | San Ambrosio | 23-Feb-13 | M6 | Exposed | -26.3493 | -79.8897 |
| Desventuradas | San Ambrosio | 23-Feb-13 | M7 | Exposed | -26.3516 | -79.8958 |
| Desventuradas | San Ambrosio | 23-Feb-13 | M8 | Exposed | -26.3505 | -79.9017 |
| Desventuradas | San Ambrosio | 20-Feb-13 | M9 | Exposed | -26.3485 | -79.9061 |
| Juan Fernández | Robinson Crusoe | 11-Jan-14 | RC1 | Protected | -33.60499 | -78.87170 |
| Juan Fernández | Robinson Crusoe | 14-Jan-14 | RC10 | Exposed | -33.65974 | -78.88843 |
| Juan Fernández | Robinson Crusoe | 16-Jan-14 | RC11 | Protected | -33.63900 | -78.81391 |
| Juan Fernández | Robinson Crusoe | 16-Jan-14 | RC12 | Protected | -33.63858 | -78.79855 |
| Juan Fernández | Robinson Crusoe | 17-Jan-14 | RC13 | Protected | -33.66371 | -78.93912 |
| Juan Fernández | Robinson Crusoe | 17-Jan-14 | RC14 | Protected | -33.65100 | -78.92200 |
| Juan Fernández | Robinson Crusoe | 17-Jan-14 | RC15 | Protected | -33.64465 | -78.90948 |
| Juan Fernández | Robinson Crusoe | 18-Jan-14 | RC16 | Protected | -33.61551 | -78.88913 |
| Juan Fernández | Robinson Crusoe | 18-Jan-14 | RC17 | Protected | -33.61401 | -78.84803 |
| Juan Fernández | Robinson Crusoe | 18-Jan-14 | RC18 | Protected | -33.65635 | -78.77660 |
| Juan Fernández | Robinson Crusoe | 11-Jan-14 | RC2 | Protected | -33.62381 | -78.83978 |
| Juan Fernández | Robinson Crusoe | 12-Jan-14 | RC3 | Protected | -33.63759 | -78.88902 |
| Juan Fernández | Robinson Crusoe | 12-Jan-14 | RC4 | Protected | -33.67308 | -78.94286 |
| Juan Fernández | Robinson Crusoe | 13-Jan-14 | RC5 | Exposed | -33.67234 | -78.86427 |

**S1Table.**

Continued

| Archipelago | Island | Date | Station | Exposure | Latitude | Longitude |
| --- | --- | --- | --- | --- | --- | --- |
| Juan Fernández | Robinson Crusoe | 13-Jan-14 | RC6 | Exposed | -33.71457 | -78.92334 |
| Juan Fernández | Robinson Crusoe | 13-Jan-14 | RC7 | Exposed | -33.69928 | -78.95551 |
| Juan Fernández | Robinson Crusoe | 14-Jan-14 | RC8 | Exposed | -33.68271 | -78.93410 |
| Juan Fernández | Robinson Crusoe | 14-Jan-14 | RC9 | Exposed | -33.66700 | -78.92028 |
