# Supplementary material for: Marine Biodiversity in Juan Fernández and Desventuradas Islands, Chile: Global Endemism Hotspots
Source: PLoS One. 2016 Jan 6;11(1):e0145059. doi: 10.1371/journal.pone.0145059 (PMC4703205; doi:10.1371/journal.pone.0145059)
Supplement: S2 Table — Resource species are those consumed directly or used as bait in the lobster and crab fisheries. (DOCX) [file pone.0145059.s002.docx]

**S2 Table. Fishes observed around San Ambrosio Island and Robinson Crusoe and Santa Clara islands.** Taxa listed in phylogenetic order. Trophic number from Fishbase [26]. Trophic: BI – benthic invertivore, Herb – herbivore, BI/P – benthic invertivore/piscivore, Pisc – piscivore, Plank – planktivore, Biogeo. (Biogeographic) affinity: DES – Desventuradas, ETP – Eastern Tropical Pacific, JF – Juan Fernández, Easter – Easter Island, NAZCA – Nazca Ridge, Indo – Indo-Pacific, Use is for resource species consumed directly (food) or used as bait in the lobster and crab fisheries.

| Family | Taxa | Common name | Trophic number | Trophic  group | Biogeo. affinity | Use |
| --- | --- | --- | --- | --- | --- | --- |
| Squalidae | *Squalus mitsukurii* | Shortspine spurdog | 4.4 | Pisc | Circumtropical |  |
| Muraenidae | *Gymnothorax australicola* | South Pacific moray | 3.8 | BI/P | South Pacific |  |
| Muraenidae | *Gymnothorax bathyphylus* | Deep-dwelling moray | 4.0 | BI/P | DES Easter |  |
| Muraenidae | *Gymnothorax porphyreus* | Lowfin Moray | 3.8 | BI/P | South Pacific | Bait |
| Congridae | *Gnathophis cf. smithi* | Smith's Conger | 3.5 | BI/P | DES JF |  |
| Ophichthidae | *Scolecenchelys chilensis* | Chilean Snake Eel | 3.9 | BI/P | DES JF |  |
| Ophichthidae | *Scolecenchelys profundorum* | Deep Snake Eel | 3.9 | BI/P | NAZCA |  |
| Synodontidae | *Synodus capricornis* | Capricorn lizardfish | 4.1 | Pisc | Antitropical |  |
| Gonorynchidae | *Gonorynchus greyi* | Grey's Sandfish | 3.4 | Plank | South Pacific |  |
| Exocoetidae | *Cheilopogon spilonotopterus* | Stained flyingfish | 3.8 | Plank | Indo |  |
| Antennariidae | *Antennatus coccineus* | Scarlet frogfish | 4.2 | Pisc | Indo |  |
| Antennariidae | *Antennatus sanguineus* | Bloody frogfish | 4.2 | Pisc | ETP |  |
| Moridae | *Lotella fernandeziana* | Morid Cod | 3.5 | BI/P | DES JF |  |
| Ophidiidae | *Ophidion metoecus* | Cusk-eel | 3.5 | BI/P | DES JF |  |
| Trachichthyidae | *Paratrachichthys fernandezianus* | Chilean sandpaperfish | 4.1 | Pisc | DES JF |  |
| Monocentridae | *Monocentris reedi* | Pinecone Fish | 3.6 | BI | DES JF |  |
| Centriscidae | *Macroramphosus scolopax* | Longspine snipefish | 3.5 | BI | Circumtropical |  |
| Centriscidae | *Macroramphosus gracilis* | Slender snipefish | 3.5 | BI | Circumtropical |  |
| Syngnathidae | *Cosmocampus heraldi* | Pipefish | 3.2 | BI | Des JF |  |
| Scorpaenidae | *Scorpaena fernandeziana* | Fernandez Scorpionfish | 3.7 | BI | DES JF |  |
| Scorpaenidae | *Scorpaena thomsoni* | Thomson Scorpionfish | 3.7 | BI | DES JF |  |
| Scorpaenidae | *Scorpaenodes englerti* | Englert's scorpionfish | 3.5 | BI | DES Easter |  |

S1 continued

| Family | Taxa | Common name | Trophic number | Trophic  group | Biogeo. affinity | Use |
| --- | --- | --- | --- | --- | --- | --- |
| Neosebastidae | *Maxillicosta reticulata* | Gunard Scorpionfish | 3.4 | BI | DES JF |  |
| Triglidae | *Pterygotrigla picta* | Spotted gurnard | 3.7 | BI | South Pacific |  |
| Polyprionidae | *Polyprion oxygeneios* | Hapuku wreckfish | 4.5 | Pisc | Circumglobal southern waters |  |
| Callanthiidae | *Callanthias platei* | Splendid Perch | 3.5 | Plank | Des JF |  |
| Serranidae | *Caprodon longimanus* | Pink maomao | 3.9 | Plank | Des JF | Bait |
| Serranidae | *Hypoplectrodes semicinctum* | Sea Perch | 4.0 | Pisc | DES JF |  |
| Serranidae | *Paralabrax* sp. |  | 4.0 | Pisc | Coastal Chile |  |
| Serranidae | *Plectranthias exsul* | Perchlet | 4.1 | Pisc | DES JF |  |
| Serranidae | *Trachypoma macracanthus* | Toadstool groper | 3.5 | BI/P | South Pacific |  |
| Carangidae | *Pseudocaranx chilensis* | Juan Fernandez trevally | 3.6 | BI/P | DES JF | Bait, food |
| Carangidae | *Seriola lalandi* | Yellowtail Amberjack | 4.1 | Pisc | Circumtropical | Bait, food |
| Kyphosidae | *Girella albostriata* | Drummer | 2.0 | Herb | DES JF | Bait |
| Kyphosidae | *Kyphosus cinerascens* | Chub | 2.0 | Herb | Indo | Food |
| Kyphosidae | *Scorpis chilensis* | Chilean Sweeper | 3.3 | Plank | DES JF | Bait, food |
| Sciaenidae | *Umbrina reedi* | King Croaker | 3.4 | BI | DES JF | Bait, food |
| Chaetodontidae | *Amphichaetodon melbae* | Narrow-barred butterflyfish | 3.2 | BI | Des JF |  |
| Pentacerotidae | *Pentaceros capensis* | Armorhead | 3.5 | BI | Circumtropical |  |
| Chironemidae | *Chironemus bicornis* | Two Horned Kelpfish | 3.5 | BI | Des JF |  |
| Chironemidae | *Chironemus delfini* | Dolphin Kelpfish | 3.5 | BI | Des JF |  |
| Cheilodactylidae | *Nemadactylus gayi* | Gay's Morwong | 3.2 | Plank | Des JF | Bait, food |
| Pinguipedidae | *Parapercis dockinsi* | Sandperch | 3.5 | BI | Des JF |  |
| Pomacentridae | *Chromis meridiana* | Chromis | 3.0 | Plank | Des |  |
| Labridae | *Pseudolabrus gayi* | Gay's Wrasse | 3.4 | BI | DES JF |  |
| Labridae | *Malapterus reticulatus* | Reticulated Wrasse | 3.5 | BI | DES JF |  |
| Labridae | *Suezichthys* sp. | Slender Wrasse | 3.3 | BI | DES JF |  |
| Blenniidae | *Entomacrodus chapmani* | Chapman's Blenny | 2.0 | Herb | DES Easter |  |

S1 continued

| Family | Taxa | Common name | Trophic number | Trophic  group | Biogeo. affinity | Use |
| --- | --- | --- | --- | --- | --- | --- |
| Blenniidae | *Scartichthys variolatus* | Spotted Rockskipper | 2.6 | Herb | DES JF |  |
| Gobiidae | *Paratrimma urospila* | Spottail Dwarfgoby | 3.3 | BI | DES JF |  |
| Gobiidae | *Paratrimma nigrimenta* | Dwarfgoby | 3.3 | BI | JF |  |
| Bothidae | *Arnoglossus coeruleosticta* | Blue Lefteye Flounder | 4.3 | Pisc | Des JF |  |
| Paralichthyidae | *Paralichthys fernandezianus* | Juan Fernandez Flounder | 4.3 | Pisc | DES JF | Food |
| Soleidae | *Aseraggodes bahamondei* | Blue Lefteye Flounder | 3.6 | BI | South Pacific |  |
